# A162 INTERLEUKIN-4 SIGNALING IS NOT A PREREQUISITE FOR HELMINTH-THERAPY PROTECTION AGAINST COLITIS

**DOI:** 10.1093/jcag/gwae059.162

**Published:** 2025-02-10

**Authors:** L Kraemer, D McKay, A Wang

**Affiliations:** University of Calgary, Calgary, AB, Canada; University of Calgary, Calgary, AB, Canada; University of Calgary, Calgary, AB, Canada

## Abstract

**Background:**

Infection with helminth parasites drives Th2 immunity, characterized by the cytokines IL-4, -5, -13, and IL-10, and expansion of regulatory cells. These orchestrated immune responses promote an immunomodulatory/immunosuppressive environment, which can protect the host from concomitant inflammation. Mice infected with the tapeworm *Hymenolepis diminuta* display a strong Th2 immune response and they are protected from DNBS-induced colitis. Surprisingly, preliminary data indicated that *il-4ra*^*-/-*^ mice (where the worm establishes) were protected from DNBS-induced colitis. Here we sought to confirm or refute the findings in *il-4ra*^*-/-*^ and, if positive, investigate the role of IL-10 and macrophages in the anti-colitic effect.

**Aims:**

To determine if IL-10 and macrophages play key roles in the protection against colitis in *il-4ra*^*-/-*^ mice infected with *H. diminuta*.

**Methods:**

BALB/c (WT) and *il-4ra*^*-/-*^ mice (IL-4ra^-/-^) (n=4-15/group) were orally infected with 5 cysticercoids of *H. diminuta* and 8 days post-infection (dpi) were treated with 2.5 mg of dinitrobenzene sulfonic acid (DNBS) to induce colitis. In some experiments, mice were treated 3 times i.p. with neutralizing anti-IL10 antibody (100 mg/mouse) or clodronate-liposomes (100 ml/mouse) to deplete macrophages (5 days and 24h before and after DNBS). Mice were necropsied 72h post-DNBS and disease was assessed through macroscopic disease activity score (DAS), colon length, weight loss, colonic MPO activity, blood leucocyte profile and histopathological score. Cytokine production by antigen-specific (*H.d.* antigen 200mg/mL; 48h) stimulated splenocytes was measured by ELISA.

**Results:**

DNBS treatment induced colitis in WT and IL-4ra^-/-^ and prior infection with *H. diminuta* was anti-colitic in both groups as shown by reduced DAS, weight loss, MPO activity, and histopathology. Compared to WT, *il-4ra*^*-/-*^ mice had less blood eosinophilia, and il-5 and il-10 production by worm-antigen stimulated splenocytes, although il-10 production was increased compared to splenocytes from uninfected control mice. Immunoneutralization of il-10 and macrophage depletion abolished the protective effects of infection with *H. diminuta*, with the clondronate-treated mice displaying severe disease.

**Conclusions:**

Despite harbouring a patient infection with *H. diminuta, il-4ra*^*-/-*^ mice are protected from DNBS-induced colitis that is, at least in part dependent on IL-10 and likely a phenotype of regulatory macrophage. The data suggest redundancy in the anti-colitic effects of *H. diminuta* infection, such that absence of il-4ra is compensated for via other, yet to be determined, mechanisms.

**Funding Agencies:**

CAG, CIHRTRIANGLE

